# The Role of Radiotherapy After Full Course of Chemotherapy in the Treatment of Diffuse Large B‐Cell Lymphoma: A Systematic Review and Meta‐Analysis

**DOI:** 10.1111/jcmm.70822

**Published:** 2025-09-19

**Authors:** Xiping Liang, Xu Deng, Kanglin Xie, Chaoyu Wang, Yao Liu

**Affiliations:** ^1^ Department of Hematology‐Oncology Chongqing Key Laboratory of Translational Research for Cancer Metastasis and Individualized Treatment Chongqing University Cancer Hospital Chongqing China; ^2^ Chongqing Medical University Chongqing China

**Keywords:** aggressive B cell NHL, consolidation radiotherapy, meta‐analysis, non‐Hodgkin lymphoma, randomised controlled trials

## Abstract

Rituximab has improved response rates and overall survival in diffuse large B‐cell lymphoma (DLBCL). Radiotherapy (RT) is an effective treatment modality for lymphomas. However, significant conceptual challenges, including the application in elderly patients, varying IPI scores and CR patients, remain regarding the current use of RT. We performed a systematic review comparing chemotherapy with RT to chemotherapy alone in patients with DLBCL. We estimated hazard ratios (HR) for OS, PFS and EFS using the proportional hazards model. Five articles involving 1364 patients met inclusion criteria. Patients undergoing consolidative RT had better OS (HR = 0.46, 95% CI 0.31–0.68), PFS (HR = 0.52, 95% CI 0.22–1.25) and EFS (HR = 0.42, 95% CI 0.20–0.90) compared to those who received no RT. But no benefit was shown in patients with achievement of CR. The protective effect of consolidation RT for patients with advanced IPI scores (HR = 0.46, 95% CI 0.31–0.68) and advanced stage (HR = 0.22, 95% CI 0.08–0.59) was shown. The consolidation RT showed a significantly longer PFS (HR = 0.50, 95% CI 0.26–0.94) but no significant benefit on OS in patients with bulky disease. There was also significantly better PFS in RT patients (HR = 0.67, 95% CI 0.49–0.92), but no significant benefit on OS in old age patients. RT act as an efficacious method for DLBCL following a full course of chemotherapy. However, no OS benefit was shown in patients with advanced IPI scores, bulky disease, CR and old age.

## 1 Introduction

Non‐Hodgkin lymphoma (NHL) refers to a group of heterogeneous malignancies originating from lymphoid cells, including mature or immature B lymphocytes, T lymphocytes or natural killer (NK) cells [1]. The global age‐standardised incidence rate of NHL in 2020, the most common hematologic malignancy, was 5.8 per 100,000 persons [2]. Comprising 35% of all non‐Hodgkin lymphomas (NHL), DLBCL is the most common aggressive lymphoma in adults [3]. The current standard therapy, mainly with the addition of rituximab into CHOP (cyclophosphamide, doxorubicin, vincristine and prednisone, R‐CHOP), cures approximately two‐thirds of patients. And numerous attempts to improve outcomes—including adding novel agents to the regimen—these results have so far failed to improve these results [4]. The considerable heterogeneity, along with variations in clinical presentation, prognosis and therapeutic response, highlights the need for more targeted and personalised treatment strategies. Radiotherapy is an effective treatment option for patients with aggressive lymphomas. It was initially used as a primary modality for various lymphomas, especially early stage, and was later used as consolidation when containing bulky disease which by itself is an independent adverse prognostic factor [5]. Radiotherapy is now commonly used in localised disease [6]. Accordingly, consolidation radiotherapy is incorporated into the first‐line treatment regimen for B‐cell lymphoma, as recommended by the European Society for Medical Oncology (ESMO) and the National Comprehensive Cancer Network (NCCN) guidelines [7]. However, significant conceptual challenges remain regarding the current use of radiotherapy outside of clinical trials. These challenges include the lack of standardised definitions for bulky disease, its application in elderly patients, and its use in patients with varying International Prognostic Index (IPI) scores. Furthermore, for patients who achieve a complete remission (CR) following chemotherapy, the guidelines recommend that follow‐up treatment should consist of either ‘observation or consolidation radiotherapy’. However, The role of consolidative RT in such patients is remains controversial in clinical practice.

Although treatment recommendations for DLBCL are often based on clinical experience, expert judgement and established guidelines, they should ideally be grounded in robust data, preferably derived from randomised controlled trials [8]. In the absence of a randomised trial on this issue, a meta‐analysis of cohort and case–control studies could offer a clearer understanding of the role of RT and provide a more reliable basis for clinical decision‐making. The aim of our study was to assess the role of consolidation RT following a full course of chemotherapy in patients with DLBCL.

## 2 Methods

We included randomised controlled clinical trials (RCTs) and retrospective studies which evaluated systemic full course of chemotherapy with RT compared with full course of chemotherapy alone in DLBCL.

### 2.1 Population

We included trials involving patients with newly diagnosed DLBCL and any stage according to the Ann Arbor classification. Patients had to be treated with a full course of systemic chemotherapy with rituximab (4 to 6 cycles for I–II stage, 6 to 8 cycles for III–IV stage) and subsequent consolidation RT or no RT; of all ages, both sexes and all ethnicities. DLBCL is defined by a clinical classification and histological criteria. We accepted the following histological classifications: Working Formulation, Kiel, REAL and WHO classification. Trials that included patients who did not achieve a full course of chemotherapy were excluded. We also excluded DLBCL patients with primary central nervous system, testicular or cutaneous involvement. Studies were excluded if they (1) involved patients with refractory disease or progressive disease, or (2) reported outcome measures solely for patients undergoing RT without including those who did not receive RT. Additional exclusion criteria included the absence of original data or incomplete reporting. The treatment response was primarily assessed according to the 2014 Lugano criteria, classified as CR, partial response (PR), stable disease (SD) or disease progression (PD).

### 2.2 Interventions

RT administered in the consolidation phase on patients in DLBCL with a full course of chemotherapy including rituximab, alkylating agents, antimetabolites, topoisomerase inhibitors, anthracyclines, glucocorticoids or monoclonal antibody regimens. There had to be no restriction on dose, frequency, intensity or duration of RT.

### 2.3 Comparator

Observation only in patients with DLBCL who have a full course of chemotherapy including rituximab, alkylating agents, antimetabolites, topoisomerase inhibitors, anthracyclines, glucocorticoids or monoclonal antibody regimens.

The chemotherapy regimens were to be the same in both arms, with equal cycles in both arms.

### 2.4 Primary Outcome

Overall survival (OS) defined as the time from entry into the clinical trial (random assignment in a phase III study) until death as a result of any cause.

### 2.5 Secondary Outcomes

- Progression‐free survival (PFS), event‐free survival (EFS).

PFS was defined as the time from the date of randomisation until the date of disease progression or death.

- EFS was defined as the time from randomisation until the first event, including local regional/distant failure or death.

### 2.6 Data Synthesis and Statistical Analysis

Two review authors (Xu Deng, Kanglin Xie) independently screened the abstracts of all studies identified for their eligibility for inclusion. If that was insufficient for a decision to be made, the full‐text article was retrieved for a full review. Any disagreements were deferred and discussed with a third review author. We assessed the risk of bias in the following domains: random sequence generation, allocation concealment, blinding of participants and personnel, blinding of outcome assessment, incomplete outcome data, selective outcome reporting and other potential sources of bias.

**FIGURE 1:**
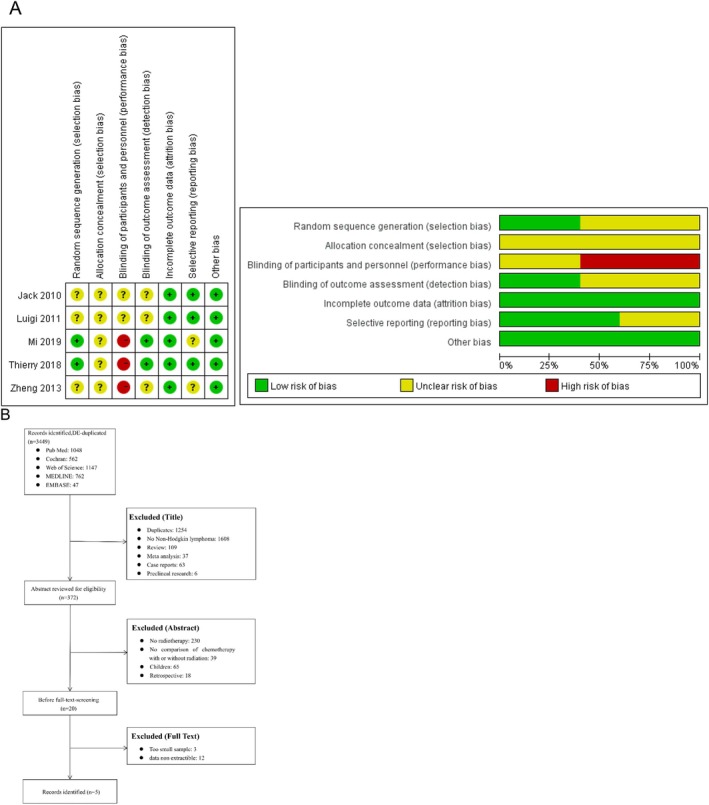
The risk of bias (A) and literature search (B).

Meta‐analysis was performed using Review Manager 5.2 software. Two review authors (Xu Deng, Kanglin Xie) independently assessed the risk of bias in the included studies and cross‐verified the results. The risk of bias evaluation was conducted using the Cochrane Handbook version 5.1.0. The risk of bias was assessed as low, high or unclear. Several studies did not report sufficiently detailed methods, making it impossible to assess the potential risk of bias. Commonly under‐reported parameters included details such as concealment of random allocation, blinding of outcome assessors and declaration of conflicts of interest. Due to the specific nature of tumours, blinding of participants or clinicians is often not feasible. Therefore, the lack of blinding in the included studies is unlikely to have affected the results. Figure 1A summarise the risk of bias findings.

For time‐to‐event variables, the treatment effect in each individual study was expressed as the hazard ratio (HR) for the radiotherapy (RT) arm compared to the no‐RT arm. The 95% confidence interval (CI) was calculated for each point estimate. The HR and 95% CI were directly extracted from the Cox proportional hazards model used in the multivariate analysis of the original studies.

## 3 Results

### 3.1 Literature Search

The literature search yielded 3449 potentially relevant published titles. After the initial review, 372 titles were potentially appropriate. Of these, 350 were excluded for the following reasons: they did not concern radiotherapy, they were not adult, they were not original studies, they did not include a no‐RT group, and there was no comparison of chemotherapy with or without radiation. After reviewing the remaining 20 studies, we excluded 3 articles which have a small sample and 12 papers which did not provide extractible data.

The flowchart of the reviews showed the detailed process of selection (Figure 1B).

### 3.2 Study Characteristics

With the aim to identify clinical trials that assessed the role of consolidation radiotherapy after a full course of chemotherapy in a randomised manner, our search revealed 5 trials including 1364 patients amenable for this meta‐analysis. The methodological characteristics of the selected trials included in this meta‐analysis are shown in Table 1. All patients were treated with R‐CHOP chemotherapy. Two articles studied the effects of chemotherapy alone or chemoradiotherapy on patients only in stage I–II patients. The other three studies analysed the effects of chemotherapy alone and chemoradiotherapy on patient prognosis in patients with advanced stage. Different studies have different definitions of combined large masses, ranging from larger than 5 cm to larger than 10 cm. There were four articles concerned with high IPI scores of the disease, and two papers included old patients. Two papers concerned with adverse events and five articles included patients with CR following a full course of treatment.

**TABLE 1:** Study Characteristics.

First author	Journal	Country of origin	Age, years (RT/no RT)	Follow‐up duration (years)	Stage	Patients, *n* (RT/no RT)	C hemotherapy	Radiation dose, GY	Definition of bulky mass	OS years (LOG)	PFS month (LOG)	EFS month (LOG)	Age > 60 years (RT/no RT)	IPI > 3 (RT/No RT)	III‐IV (RT/No RT)	CR (RT/No RT)
Jack Phan	J Clin Oncol. 2010	USA	61	3	I–IV	142/327	RCHOP	—	> 5 cm	1.6601	1.1394	—	Yes	21/96	39/240	139/208
Mi Joo Chung	J Radiat Res. 2019	Korea	54	3.5	I–II	92/184	RCHOP	36	> 7.5 cm	0.4463	0.2459	—	61/28	3/7	—	—
Zheng Shi	Int J Radiat Oncol Biol Phys. 2013	USA	59.4	2.7	III–IV	14/96	RCHOP	30–40.8	> 5 cm	—	—	—	—	6/55	14/96	14/69
Luigi Marcheselli	Leuk Lymphoma. 2011	Italy	69	2.5	I–IV	40/142	RCHOP	34	> 6 cm	0.8210	—	1.2730	—	10/63	19/100	—
Thierry Lamy	Blood. 2018	France	—	5.3	I–II	164/163	RCHOP	40	> 7 cm	—	—	0.4943	—	—	—	144/137

### 3.3 Association of RT and Efficacy

**FIGURE 2:**
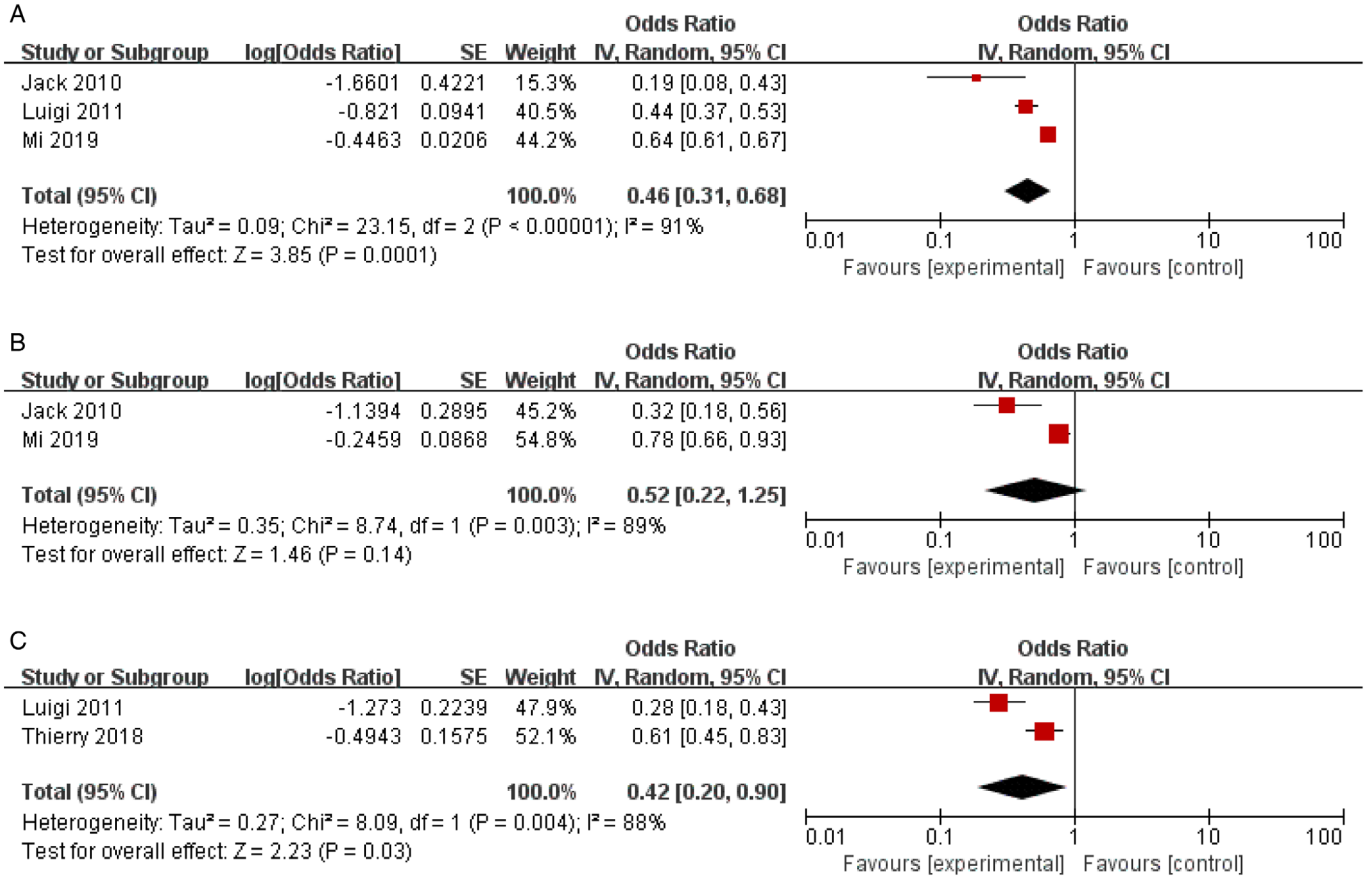
Effect of consolidation radiotherapy on overall survival (A), PFS (B) and EFS (C).

To evaluate the efficacy of consolidation RT and systemic chemotherapy alone for DLBCL, we synthesised data on OS, PFS and EFS. Three of five articles with a total of 927 patients contributed to the analysis of the OS [9, 10, 11]. Notably, patients undergoing chemoradiotherapy displayed a significantly better OS compared to no RT (RT vs. no‐RT: HR = 0.46, 95% CI 0.31–0.68, *p* = 0.0001; Figure 2A), with heterogeneity among studies (*p* < 0.00001, *I* ^2^ = 91%). Two articles including 745 patients were described in the analysis of PFS. While similar PFS was obtained in all patients (RT vs. no‐RT: HR = 0.52, 95% CI 0.22–1.25, *p* = 0.14; Figure 2B). Two studies comprising 511 patients were analysed for EFS [11, 12]. The consolidation RT showed an improvement in EFS (HR = 0.42, 95% CI 0.20–0.90, *p* = 0.03; Figure 2C). Both PFS and EFS had heterogeneity among studies (*p* < 0.05, *I* ^2^ > 50%).

**FIGURE 3:**
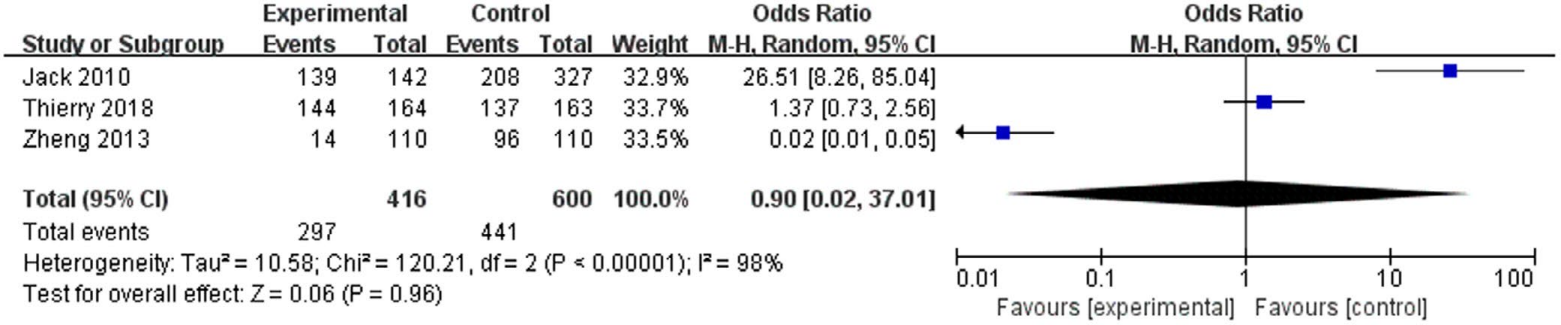
Effect of consolidation radiotherapy on CR patients.

As consolidation radiotherapy is part of the first‐line treatment of non‐Hodgkin B‐cell lymphoma who achieve ≥PR after chemotherapy, radiotherapy can be added as appropriate treatment including giant mass, according to the guidelines. In this meta‐analysis, a total of 3 articles totalling 906 patients with CR following a full course of chemotherapy were included, which showed no benefit to CR patients compared to no RT (HR = 0.90, 95% CI 0.02–37.01, *p* = 0.96; Figure 3), with the heterogeneity among studies (*p* < 0.05, *I* ^2^ = 97%).

### 3.4 Association of Population and RT

**FIGURE 4:**
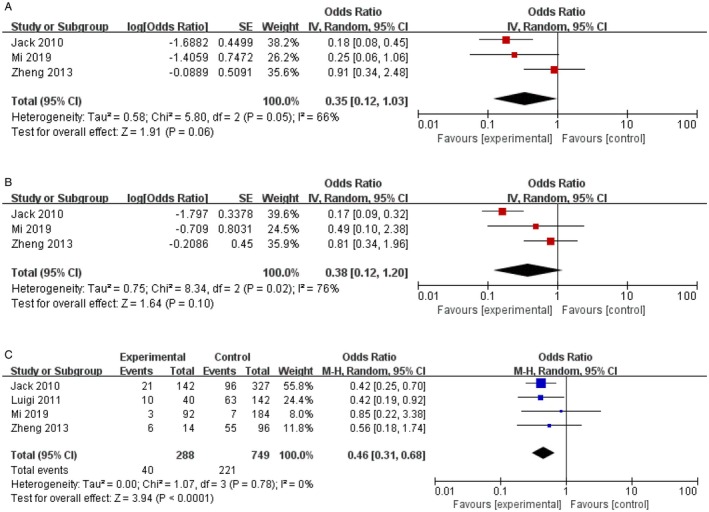
Effect of consolidation radiotherapy on OS (A) and PFS (B) in patients with advance IPI score, and the protective effect of consolidation radiotherapy for patients with advance IPI scores (C).

Four studies comprising 261 patients with advanced IPI score (IPI ≥ 3 score) [10, 11, 13, 14], but three studies were analysed for OS and PFS. The consolidative of RT showed a trend of improvement in OS in all patients (RT vs. no‐RT: HR = 0.35, 95% CI 0.12–1.03, *p* = 0.06; Figure 4A), with no heterogeneity among studies (*p* = 0.05). The RT arm was associated with no significant improvement in PFS (RT vs. no‐RT: HR = 0.38, 95% CI 0.12–1.2, *p* = 0.1; Figure 4B), with heterogeneity among studies (*p* = 0.02, *I* ^2^ = 76%). However, there was a protective effect of consolidation radiotherapy for patients with advanced IPI scores (HR = 0.46, 95% CI 0.31–0.68, *p* < 0.0001; Figure 4C). No publication bias was detected with a *p* value of 0.78 in Egger's test and no significant outcome of influence analysis was observed.

**FIGURE 5:**
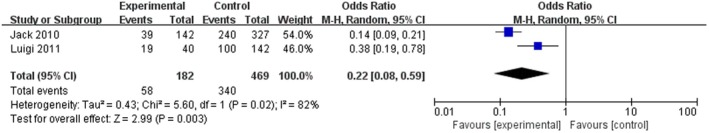
Effect of consolidation radiotherapy on advance stage patients.

A total of 651 patients were referred in two articles. The protective effect of RT for patients with advanced stage was shown in Figure 5 (HR = 0.22, 95% CI 0.08–0.59, *p* = 0.003), with heterogeneity among studies (*p* = 0.02, *I* ^2^ = 82%). We did not show RT benefit on OS and PFS because of incomplete data.

**FIGURE 6:**
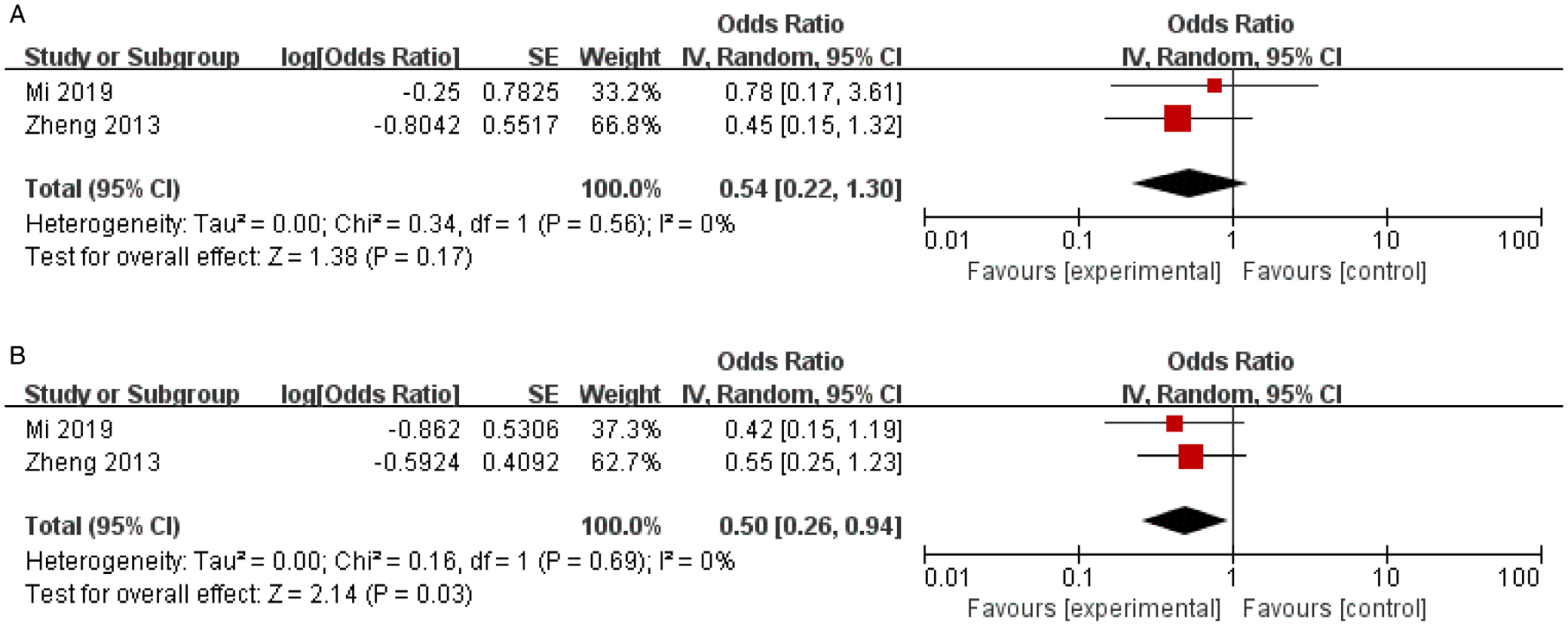
Effect of consolidation radiotherapy on OS (A) and PFS (B) in patients with bulky disease.

Two studies with bulky disease were analysed for OS and PFS. The RT arm had a trend of improving the OS, but no significant difference (RT vs. no‐RT: HR = 0.54, 95% CI 0.22–1.30, *p* = 0.17; Figure 6A). However, the consolidation RT showed a significantly longer PFS (RT vs. no‐RT: HR = 0.50, 95% CI 0.26–0.94, *p* = 0.03; Figure 6B), with no heterogeneity among studies (*p* = 0.69, *I* ^2^ = 0%).

**FIGURE 7:**
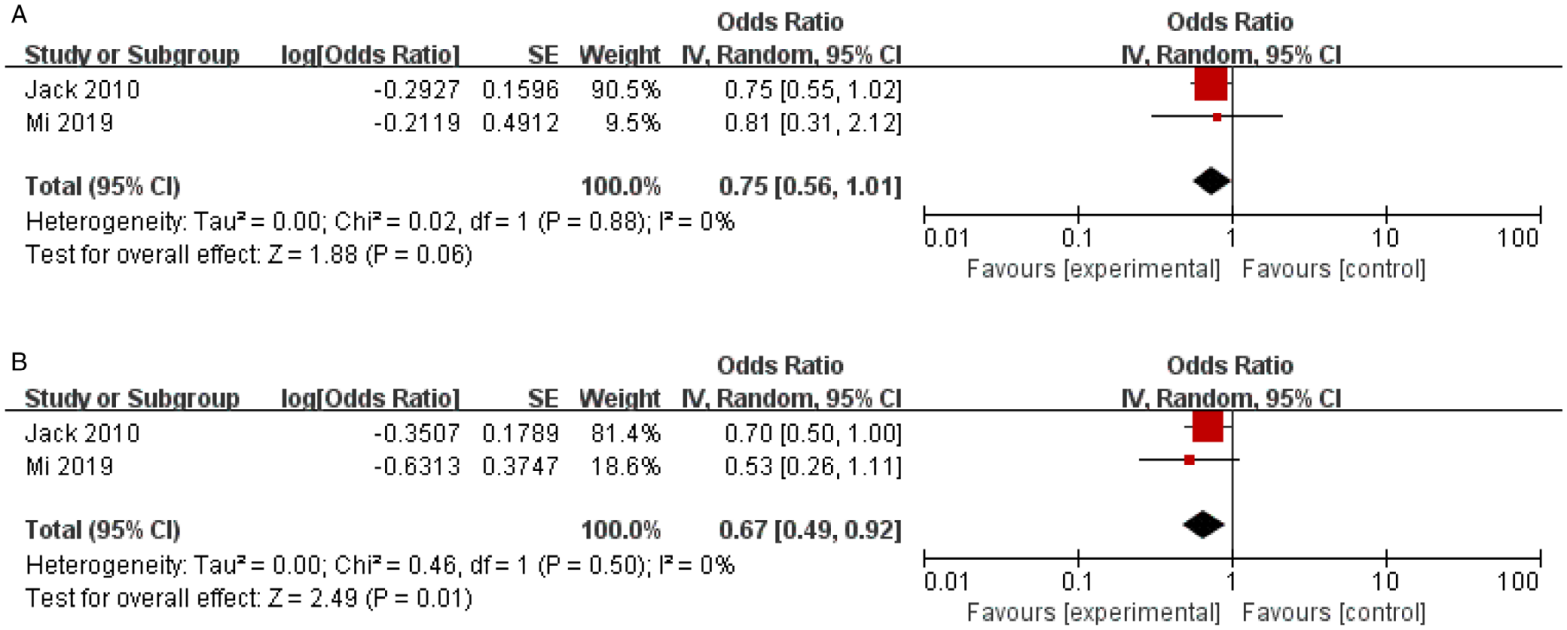
Effect of consolidation radiotherapy on OS (A) and PFS (B) in old patients.

Since age is regarded as a key prognostic factor for DLBCL, elderly patients benefit from consolidation radiotherapy remains controversial. Two trials had an age of over 60 years contributing to the analysis of OS and PFS. The consolidation radiotherapy had a trend of improving the OS, but no significant difference (RT vs. no‐RT: HR = 0.75, 95% CI 0.56–1.01, *p* = 0.06; Figure 7A), with no heterogeneity among studies (*p* = 0.88, *I* ^2^ = 0%). However, there was significantly better PFS in consolidation radiotherapy patients (RT vs. no‐RT: HR = 0.67, 95% CI 0.49–0.92, *p* = 0.01; Figure 7B), with no heterogeneity among studies (*p* = 0.5, *I* ^2^ = 0%).

### 3.5 Adverse Events and Heterogeneity

We did not show the side effect of radiotherapy on patients because of incomplete data. Given the significant heterogeneity, we analysed the data by using the following stratifications: (i) the applied chemotherapy was similar in both arms, (ii) whether the same course of RCHOP was used, (iii) the dose of radiotherapy and (iv) whether the radiotherapy dose was given only in complete morphologic remission. We failed to explain between‐trial heterogeneity by stratifying on early phase, youth patients and low IPI score of these subgroups.

## 4 Discussion

We here provide a large and comprehensive meta‐analysis with the best currently available data from RCTs and retrospective studies on consolidation RT in the treatment of DLBCL. In summary, we find that consolidation RT has a significant benefit on OS and EFS for people with DLBCL compared to chemotherapy following a full course of treatment. The protective effect of consolidation RT for patients with advance stage and high IPI score was shown. People with bulky disease and elderly age obtain a longer PFS in consolidation RT patients compared to chemotherapy alone. These trials were unable to conclusively determine the benefit of RT for patients with CR following the full course of treatment. Due to incomplete reporting of early stage, youth age, low IPI score and PR patients, we were not able to find a survival benefit for these patients.

Radiotherapy is an effective treatment option for patients with aggressive lymphomas. Radiotherapy was first used as a primary treatment for lymphomas and later became a consolidation therapy with the advent of anthracycline regimens and is now commonly used for localised disease and was included in the first‐line treatment to ESMO and NCCN guidelines [5, 6, 7]. Early‐stage diseases are usually treated with combination chemotherapy followed by radiation of affected sites and generally have good outcomes [14, 15, 16, 17]. However, about 60%–70% of people with DLBCL present in advanced stages of the disease [18, 19]. Although rituximab has significantly improved the prognosis of DLBCL, patients with advanced stage still have a high risk of disease relapse and death [20, 21]. This presents a challenging treatment paradigm to the treating clinician. Two conclusions are evident from the RT patterns of failure in these patients. First, RT achieved local control at the original disease site when used in combination with abbreviated chemotherapy, and second, abbreviated chemotherapy failed to control disease at distant sites and thus was responsible for an inferior outcome [22]. RT following abbreviated‐course chemotherapy (i.e., three cycles), not full‐course chemotherapy, might contribute more to local control and survival in Stage I to II and non‐bulky DLBCL. Full‐course R‐CHOP over six cycles would obviate the additional impact of local disease control in the R‐CHOP plus consolidative RT group [11]. The use of a full course chemotherapy would control systemic disease but would still not eliminate the need for RT [23]; indeed, our meta‐analysis provides further evidence that even though advanced stage patients received a full course of chemotherapy, a protective effect of consolidation RT for patients with advanced stage from RT was still evidenced. However, due to missing data, we are unable to assess the survival benefit of RT and need more study to explore this issue.

IPI score known as prognostic factors was associated with worse OS and PFS, and whether radiation therapy improves survival for such patients is controversial. Four articles [9, 10, 11, 13] showed the protective effect of consolidation radiotherapy for patients with advanced IPI scores but no benefited from RT on OS and PFS. There were some reasons for it. First, RT can locally control tumours and help reduce the risk of local recurrence but cannot target systemic disease. Second, high IPI score patients are often combined with poor physical status. Patients with local radiotherapy may be intolerant to side effects, which may affect the survival of patients. Whatmore, the lack of significant benefit of radiation therapy in terms of both OS and PFS may be attributed to the small sample size and tumour heterogeneity. However, further research is needed to explore this issue.

A previous study reported on the role of involved‐field RT in patients with PR, showing that it yields comparable outcomes to salvage chemotherapy [24]. Most of the study revealed beneficial outcomes in patients who achieved PR after chemotherapy; Thierry Lamy et al. showed that CR was documented in most PR patients after additional cycles of R‐CHOP and RT [12]. But no benefit was shown in patients with CR after the full course of chemotherapy combined with RT. Unfortunately, we could not evaluate the potential role of RT in patients who achieved PR because, according to our institutional guidelines, once PR is documented, then salvage chemotherapy is given; hence, less data was measured.

The presence of bulky disease is perceived by most clinicians as a reason to deliver RT. Consolidation RT in patients with bulky disease had favourable effects on PFS and OS according to existing data. Mondello et al. showed a highly significant advantage in PFS in patients receiving RT [25]. Data from that study will be instructive and lend further credence to the use of consolidative RT in advanced aggressive NHL. However, Jack Phan et al. revealed that bulky disease status did not affect outcomes and patients with and without bulky disease benefited equally from RT [9]. In this meta‐analysis, the consolidation of RT showed a significantly longer PFS but no benefit on OS in patients with bulky disease. More studies are needed in conjunction with personalised therapy approaches to evaluate this modality.

With the rising incidence of NHL and increasing life expectancy of survivors, the role of consolidation RT needs to be re‐evaluated in the rituximab era. In the rituximab era, several trials for R‐CHOP chemotherapy alone in elderly patients with DLBCL showed drastic improvement in survival. This has brought about the question of whether consolidative RT after R‐CHOP is still necessary or not [26, 27, 28]. Improved survival with the addition of consolidative RT following the full course of chemotherapy in DLBCL in the rituximab era in our data. More longer PFS was also shown in elderly DLBCL patients, which is consistent in subgroup analyses of elderly patients, including Jack et al. and Mi Joo et al. study [9, 10]. The addition of consolidative RT also improves PFS in our reviews in elderly patients.

The optimal radiation dose remains to be determined. It has been shown that a dose greater than 40 Gy provides better local control compared to doses below 40 Gy; however, this finding is somewhat intuitive, and the long‐term effects must also be considered [29]. Lowry et al. reported that no significant difference was observed between the group receiving 30 Gy and those receiving 40–45 Gy in terms of freedom from progression, PFS and OS in aggressive non‐Hodgkin lymphoma (NHL) [30]. Our review considers RT doses ranging from 30 to 40 Gy. For early‐stage, radiation dose was not a significant factor for OS after full course of chemotherapy [10]. However, patients with advanced stage had a survival benefit from RT doses ranging from 40 to 50 Gy [31]. RT doses ≥ 40 Gy serve as an independent prognostic factor for residual disease treatment, at the cost of increased pulmonary toxicity [31]. Further studies are needed to identify the optimal RT dose for the treatment of NHL.

### 4.1 Limitation

Our review has several limitations as both trials differ in their patient populations. Not all trials were performed as the same radiation dose and the same number of cycles of chemotherapy became part of the standard of care. Due to the inclusion criteria of a full course of chemotherapy, less data was included. In addition, we did not compare low IPI, youths' patients, early stage because of data scarcity. Different the length of follow‐up, it is likely that some lost to follow‐up or withdrawal may have occurred.

## 5 Conclusion

RT is an efficacious method for treating DLBCL. Consolidation RT has a better OS and EFS in people with DLBCL following a full course of chemotherapy compared to chemotherapy alone. However, patients with CR following a full course of treatment had no survival benefit from RT. The protective effect of consolidation RT for patients with advanced stage and high IPI score were shown. People with bulky disease and elderly age obtain a PFS benefit from consolidation RT. Further studies are needed to identify the RT for the treatment of these patients.

## Author Contributions

**Xiping Liang:** writing – original draft (lead), writing – review and editing (equal). **Xu Deng:** formal analysis (equal), software (equal). **Kanglin Xie:** data curation (equal), formal analysis (equal), investigation (equal). **Chaoyu Wang:** data curation (equal), formal analysis (equal), software (equal). **Yao Liu:** funding acquisition (equal), investigation (equal), methodology (equal).

## Disclosure

All authors have read and approved the manuscript. We have no financial disclosures. We are not using any copyrighted information, patient photographs, identifiers or other protected health information in this paper. No text, text boxes, figures or tables in this article have been previously published or owned by another party. Content: All figures and tables included are original content developed by the authors.

## Conflicts of Interest

The authors declare no conflicts of interest.

## Data Availability

All figures and tables included are original content developed by the authors. All data that support the findings of this study are included in this manuscript.
